# Relationship Between Mitochondrial Biological Function and Breast Cancer: An Approach Based on Mendelian Randomization Analysis

**DOI:** 10.1155/2024/4434466

**Published:** 2024-11-06

**Authors:** Shichen Miao, Qichao Ni, Jun Fang

**Affiliations:** Department of Thyroid and Breast Surgery, Affiliated Hospital of Nantong University, Medical School of Nantong University, Nantong 226001, China

**Keywords:** breast cancer, genomewide association studies, Mendelian randomization, mitochondrial function

## Abstract

**Objective:** This study aims to investigate the potential causal link between mitochondrial function and breast cancer using the Mendelian randomization (MR) analysis.

**Methods:** The data used for this study were obtained from genomewide association studies (GWAS) databases on mitochondrial biological function and breast cancer. Mitochondrial function was considered the exposure variable, breast cancer the outcome variable, and specific single nucleotide polymorphisms (SNPs) were selected as instrumental variables (IVs). Two MR methods, inverse variance weighting (IVW) and MR-Egger regression, were used to assess the causal association between mitochondrial function and breast cancer. Data analysis and visualization were performed using R software.

**Results:** The results of the analysis revealed that several genes, including 39S ribosomal protein L34, pyruvate carboxylase, rRNA methyltransferase 3, and cytochrome c oxidase assembly factor 3 homolog, are causally linked to an increased risk of breast cancer in European populations. In addition, cytochrome c oxidase subunit 8A and ADP-ribose pyrophosphatase were found to be protective factors against breast cancer in European populations. In East Asian populations, 39S ribosomal protein L52, ATP synthase subunit beta, and pyruvate dehydrogenase (acetyl-transferring) were identified as causal risk factors for breast cancer. Conversely, 39S ribosomal protein L32, ADP-ribose pyrophosphatase, and cytochrome c oxidase subunit 8A were identified as protective factors against breast cancer in this population.

**Conclusion:** In conclusion, this study provides evidence of a causal relationship between mitochondrial function and breast cancer in both European and East Asian populations. Additional research is warranted to further elucidate the mechanisms underlying this association.

## 1. Introduction

Breast cancer poses a significant threat to women's health as one of the most prevalent malignant tumors globally. Accounting for approximately 30% of female cancer cases, breast cancer also has a high mortality rate of up to 15%. In China, breast cancer has emerged as a primary contributor to female malignancies [1, 2]. Current treatment strategies for breast cancer typically involve a comprehensive approach that integrates multidisciplinary diagnostics and treatments, with surgery at the forefront and radiation therapy, chemotherapy, and various pharmaceutical interventions serving as supporting therapies [3]. However, the inherent characteristics of breast cancer, including drug resistance, recurrence, and metastasis, underscore the critical need for in-depth exploration of its underlying mechanisms of onset and progression [4]. Identifying novel therapeutic targets and advancing more effective treatment modalities are crucial areas requiring immediate attention in contemporary breast cancer research. Through the utilization of Mendelian randomization (MR) and genomewide association studies (GWAS), this research sheds light on the intricate relationship between mitochondrial function and the development of breast cancer.

Mitochondria, specialized organelles present in the majority of eukaryotic cells, are enclosed by two membranes and play a pivotal role in generating adenosine triphosphate (ATP), a crucial high-energy phosphate molecule within the cell [5]. With their unique genetic material and sophisticated system, mitochondria participate in essential cellular processes such as cell differentiation, intercellular communication, and programmed cell death known as apoptosis. They also possess the capacity to modulate cell growth and the cell cycle. In breast tumor cells, mitochondrial function commonly becomes dysregulated, resulting in mitochondrial damage and irregularities. This disrupted state impairs oxidative phosphorylation levels while promoting aerobic glycolysis [6, 7]. As a consequence, aggressive breast cancer cells actively support mitochondrial respiration to boost ATP production by mechanisms involving exaggerated expression of PGC-1*α* and amplified mitochondrial biogenesis [8].

Conventional epidemiological studies often encounter challenges related to unmeasured confounders and reverse causality, which can impede the ability to establish causal relationships between exposure and outcomes [9]. In response to these limitations, the MR research methodology was developed, originally introduced by Katan in 1986 [10]. Leveraging Mendel's second law of inheritance, MR utilizes data from GWAS to model the association between risk factors and outcomes by utilizing genetic variants as instrumental variables (IVs) [11]. By employing this approach, the method effectively mitigates the influence of confounding variables and addresses issues of reverse causation, thereby enabling the determination of causal relationships between variables [12]. In the context of this study, we utilized MR analysis with GWAS data to explore the potential causal link between mitochondrial biological function and breast cancer.

## 2. Materials and Methods

### 2.1. Data Selection

In this study, genetic data from GWAS pertaining to mitochondrial biological functions were utilized as the exposure variables, while single nucleotide polymorphisms (SNPs) strongly associated with breast cancer were chosen as IVs. The investigation encompassed breast cancer–related data as the outcome variables, examining populations from both European and East Asian backgrounds. Furthermore, the study aimed to delve into the causal link between mitochondria and different subtypes of breast cancer, specifically focusing on estrogen receptor-positive (ER+) and estrogen receptor-negative (ER−) breast cancer.

### 2.2. Data Sources

Data related to mitochondrial functions and breast cancer GWAS were sourced from https://gwas.mrcieu.ac.uk/, https://www.ebi.ac.uk/gwas/, and https://www.finngen.fi/en. Through a systematic search, a total of 89 datasets pertaining to mitochondria were identified. The European population breast cancer GWAS dataset (ebi-a-GCST90018799) consisted of 257,730 individuals with 24,133,589 SNPs, while the East Asian population breast cancer GWAS dataset (ebi-a-GCST90018656) included 79,550 participants with 12,429,464 SNPs. For ER+ breast cancer GWAS (ieu-a-1127), the sample size was 175,475 individuals with 10,680,257 SNPs, and for ER− breast cancer GWAS (ieu-a-1128), the cohort comprised 127,442 subjects with 10,680,257 SNPs (Table 1).

### 2.3. Correlation Analysis

**Figure 1:**
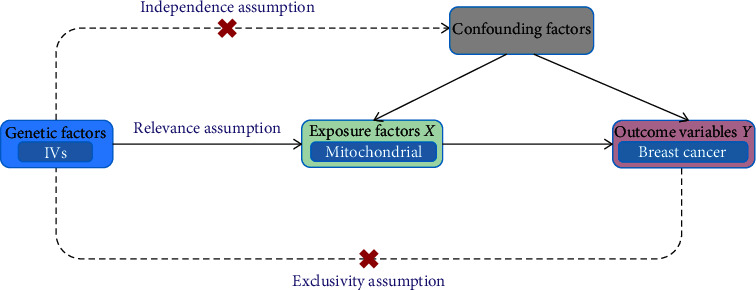
Mendelian randomization principle.

MR analysis satisfied three assumptions: (1) There was a strong correlation between the IVs and mitochondrial function (exposure factors) and (2) the IVs are independent of other confounding factors influencing breast cancer, ensuring that any associations observed are not biased by these factors. Furthermore, the IVs are posited to affect breast cancer exclusively through mitochondrial function, as illustrated in Figure 1 [13]. SNPs strongly associated with folic acid–related products were selected as IVs, using a filtering threshold of *p* < 5*e* − 06. The selection of SNPs associated with folic acid metabolism is justified by its critical role in mitochondrial function, affecting DNA methylation, synthesis pathways, energy production, ROS management, and apoptosis regulation, thus potentially altering cellular health and disease susceptibility.

### 2.4. Linkage Disequilibrium (LD)

LD describes the tendency for nearby genetic variants on the genome to be inherited together. This increases the likelihood of alleles from different loci appearing together on one chromosome compared to random occurrence [14]. Screening criteria were as follows: (1) kb > 10,000 and (2) *r*^2^ < 0.001. Kb measures the extent of LD, while *r*^2^ ranges from 0 to 1, where *r*^2^ = 1 indicates complete LD and *r*^2^ = 0 indicates a complete random assortment of SNPs. The *F*-statistic evaluates IV strength. An *F*-value < 10 defines a weak IV, while an *F*-value > 10 defines a strong IV [15–17].

### 2.5. MR Analysis

Six methods were employed to establish the causal relationship between mitochondrial exposure factors and breast cancer outcomes. These methods include inverse variance weighting (IVW), MR-Egger regression, simple mode, weighted mode, simple median, and weighted median. The TwoSampleMR package in R was utilized to visualize MR results, including scatter and forest plots and sensitivity analysis plot. The IVW method is mainly used to assess the reliability of MR analysis results, with *p* < 0.05 indicating a positive outcome [18].

### 2.6. Sensitivity Analysis

Heterogeneity was evaluated using IVW and MR-Egger tests, with *p* < 0.05 indicating significant heterogeneity. The presence of pleiotropy, where IVs affect outcomes through factors other than the exposure, was assessed using the MR-Egger intercept test, with *p* < 0.05 suggesting potential pleiotropy [19]. Sensitivity analysis utilized the “leave-one-out” method to evaluate SNP stability and detect outliers [20]. Funnel plots generated in R were used to evaluate SNP symmetry and confirm result reliability.

### 2.7. SMR Analysis

The SMR analysis leverages genetic variability as an instrumental tool to pinpoint genes linked to complex diseases through pleiotropy. This technique amalgamates data from GWAS and expression quantitative trait loci (eQTL) to establish connections between gene activity and traits, thereby identifying potential causative genes implicated in conditions such as breast cancer. The breast cancer–associated GWAS information was extracted from https://www.ebi.ac.uk/gwas/, encompassing populations of both European and East Asian descent. In addition, eQTL data were acquired from https://yanglab.westlake.edu.cn/data/SMR/GTEx_V8_cis_eqtl_Summary.html/.

### 2.8. Statistical Methods and Software

This study uses the R 4.3.1 software (https://cloud.r-project.org/) and Strawberry Perl 5.32.1.1 software (https://strawberryperl.com/) to analyze the relationship between MR. A *p* value of less than 0.05 was deemed statistically significant. The R packages “VariantAnnotation,” “gwasglue,” “dplyr,” and “tidyr” were used for the selection of SNPs strongly associated with exposure and outcome factors, applying a filter of *p* < 5*e* − 05.

## 3. Results

### 3.1. Screening the Dataset

**Figure 2:**
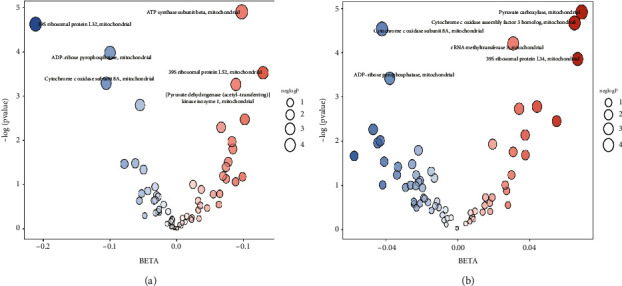
(a) The GWAS dataset comprises SNPs demonstrating a robust association with both mitochondrial function and breast cancer in the European population and (b) the GWAS dataset encompasses SNPs displaying a significant correlation with mitochondrial function and breast cancer in the East Asian population.

A total of six strongly associated SNPs with breast cancer in the European population dataset ebi-a-GCST90018799 were selected from the following mitochondrial-related components: prot-a-1943 (39S ribosomal protein L34), prot-a-2190 (pyruvate carboxylase), prot-a-641 (cytochrome c oxidase subunit 8A), prot-a-2129 (ADP-ribose pyrophosphatase), prot-a-2575 (rRNA methyltransferase 3), and prot-a-612 (cytochrome c oxidase assembly factor 3 homolog) (Figure 2(a) and Table 2). Specifically, 9 SNPs in prot-a-1943, 10 SNPs in prot-a-641, 12 SNPs in prot-a-612, 13 SNPs in prot-a-2190, 17 SNPs in prot-a-2129, and 18 SNPs in prot-a-2575 showed significant associations with breast cancer in European populations. Similarly, a collection of six SNP datasets strongly correlated with breast cancer in the East Asian population within the ebi-a-GCST90018579 dataset which included the following: prot-a-1941 (39S ribosomal protein L32), prot-a-1944 (39S ribosomal protein L52), prot-a-203 (ATP synthase subunit beta), prot-a-2129 (ADP-ribose pyrophosphatase), prot-a-2235 ([pyruvate dehydrogenase (acetyl-transferring)] kinase isozyme 1), and prot-a-641 (cytochrome c oxidase subunit 8A) (Figure 2(b) and Table 2). Among these, 4 SNPs in prot-a-1941, 8 SNPs in prot-a-1944, 10 SNPs in prot-a-203, 14 SNPs in prot-a-2129, and 4 SNPs each in prot-a-2235 and prot-a-641 exhibited strong associations with breast cancer in European populations. The F-value computation and elimination of confounders for the identified SNPs were carried out using the R package “MendelianRandomization,” with no weak IVs or confounders detected in the analysis (Figure 2 and Table 3).

### 3.2. Results of MR Analysis

To investigate the causal connection between exposure factors and outcomes, we utilized techniques such as IVW, MR-Egger regression, and two additional methods. Employing the TwoSampleMR package in R, we visualized the MR outcomes through scatterplots and forest plots. An SNP is categorized as a risk factor for breast cancer if the odds ratio (OR) surpasses 1 and the *p* value is under 0.05. Conversely, an SNP is identified as a protective factor if the OR is below 1 and the *p* value is less than 0.05.

**Figure 3:**
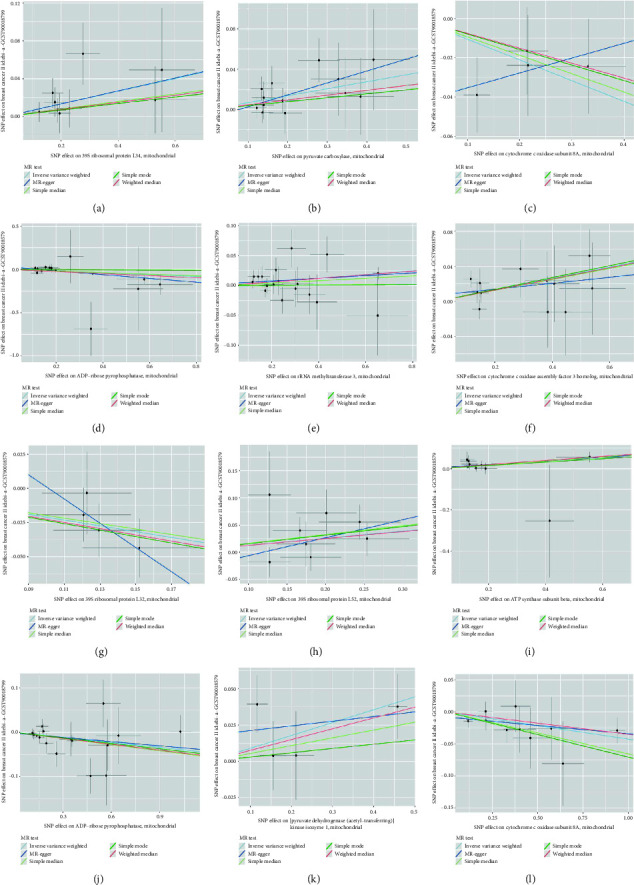
(a) Scatter plot illustrating the causal association between 39S ribosomal protein L34 and breast cancer, assessed primarily through the IVW method; (b) scatter plot demonstrating the causal link between pyruvate carboxylase and breast cancer, primarily evaluated using the IVW method; (c) scatter plot presenting the causal connection between cytochrome c oxidase subunit 8A and breast cancer, primarily assessed through the IVW method; (d) scatter plot showcasing the causal relationship between ADP-ribose pyrophosphatase and breast cancer, predominantly evaluated using the IVW method; (e) scatter plot depicting the causal association between rRNA methyltransferase 3 and breast cancer, assessed primarily through the IVW method; (f) scatter plot illustrating the causal link between cytochrome c oxidase assembly factor 3 homolog and breast cancer, primarily evaluated using the IVW method; (g) scatter plot showcasing the causal association between 39S ribosomal protein L32 and breast cancer, assessed predominantly through the IVW method; (h) scatter plot illustrating the causal relationship between 39S ribosomal protein L52 and breast cancer, primarily evaluated using the IVW method; (i) scatter plot demonstrating the causal connection between ATP synthase subunit beta and breast cancer, assessed predominantly through the IVW method; (j) scatter plot showcasing the causal link between ADP-ribose pyrophosphatase and breast cancer, primarily evaluated using the IVW method; (k) scatter plot depicting the causal association between [pyruvate dehydrogenase (acetyl-transferring)] kinase isozyme 1 and breast cancer, primarily assessed through the IVW method; and (l) scatter plot illustrating the causal relationship between cytochrome c oxidase subunit 8A and breast cancer, predominantly evaluated through the IVW method.

**Figure 4:**
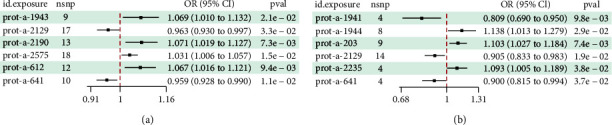
(a) Forest plot illustrating the causal associations between mitochondrial factors and breast cancer in European populations (IVW method) and (b) forest plot displaying the causal associations between mitochondrial factors and breast cancer in East Asian populations (IVW method).

The findings revealed significant causal links between specific genes and breast cancer risk in the European population. 39S ribosomal protein L34 was identified as a risk factor for breast cancer (IVW, *p*=0.021, OR = 1.069, 95% CI: 1.010–1.132), while pyruvate carboxylase showed a causal association with breast cancer as a risk factor (IVW, *p*=0.007, OR = 1.071, 95% CI: 1.019–1.127). Conversely, cytochrome c oxidase subunit 8A was associated with breast cancer as a protective factor (IVW, *p*=0.059, OR = 0.963, 95% CI: 0.928–0.990) and ADP-ribose pyrophosphatase exhibited a causal association as a protective factor against breast cancer (IVW, *p*=0.011, OR = 0.962, 95% CI: 0.930–0.997). In addition, rRNA methyltransferase 3 was linked to breast cancer as a risk factor (IVW, *p*=0.015, OR = 1.031, 95% CI: 1.006–1.057), while cytochrome c oxidase assembly factor 3 homolog was identified as a protective factor against breast cancer (IVW, *p*=0.009, OR = 1.067, 95% CI: 1.016–1.121) (Figures 3 and 4 and Table 4).

In the East Asian population, 39S ribosomal protein L32 was found to be causally associated with breast cancer as a protective factor (IVW, *p*=0.010, OR = 0.809, 95% CI: 0.690–0.950). Conversely, 39S ribosomal protein L52 was identified as a risk factor for breast cancer (IVW, *p*=0.029, OR = 1.138, 95% CI: 1.013–1.279). A causal association was observed between ATP synthase subunit beta and breast cancer as a risk factor (IVW, *p*=0.048, OR = 1.084, 95% CI: 1.027–1.184). In addition, ADP-ribose pyrophosphatase was determined to be causally associated with breast cancer as a protective factor (IVW, *p*=0.019, OR = 0.905, 95% CI: 0.833–0.983), while a causal relationship was established between [pyruvate dehydrogenase (acetyl-transferring)] kinase isozyme 1 and breast cancer as a risk factor (IVW, *p*=0.038, OR = 1.093, 95% CI: 1.005–1.189). Furthermore, cytochrome c oxidase subunit 8A was found to be causally associated with breast cancer as a protective factor (IVW, *p*=0.037, OR = 0.900, 95% CI: 0.815–0.994) (Figures 3 and 4 and Table 4).

### 3.3. MR Results for Breast Cancer Subtypes

The results demonstrated a causal relationship between 39S ribosomal protein L33 and ER+ breast cancer, highlighting its role as a protective factor (IVW, *p*=0.0015, OR = 0.965, 95% CI: 0.943–0.986). Moreover, the analysis revealed that *N*-acetylglutamate synthase is causally associated with ER+ breast cancer, identified as a risk factor (IVW, *p*=0.025, OR = 1.060, 95% CI: 1.008–1.116). In addition, NADH dehydrogenase [ubiquinone] iron-sulfur protein 4 was found to be causally linked to ER+ breast cancer as a risk factor (IVW, *p*=0.031, OR = 1.017, 95% CI: 1.002–1.033). Furthermore, [pyruvate dehydrogenase (acetyl-transferring)] kinase isozyme 1 was identified as a protective factor against ER+ breast cancer (IVW, *p*=0.0078, OR = 0.960, 95% CI: 0.931–0.989).

For ER− breast cancer, the analysis highlighted hydroxymethylglutaryl-CoA synthase as a protective factor, showing a causal association with breast cancer (IVW, *p*=0.044, OR = 0.935, 95% CI: 0.875–0.998). Conversely, mitochondrial glutamate carrier 2 was found to be causally associated with ER-breast cancer as a risk factor (IVW, *p*=0.049, OR = 1.083, 95% CI: 1.000–1.174), along with mitochondrial sodium/hydrogen exchanger 9B2 which was identified as a risk factor for ER-breast cancer (IVW, *p*=0.031, OR = 1.074, 95% CI: 1.006–1.145). Furthermore, the essential MCU regulator was causally linked to ER-breast cancer as a risk factor (IVW, *p*=0.0077, OR = 1.071, 95% CI: 1.018–1.127), while ES1 protein homolog emerged as a protective factor against breast cancer (IVW, *p*=0.035, OR = 0.943, 95% CI: 0.893–0.996). The analysis also revealed that mitochondrial import inner membrane translocase subunit TIM14 is causally associated with ER− breast cancer, acting as a risk factor (IVW, *p*=0.045, OR = 1.071, 95% CI: 1.002–1.145) (Table 5).

### 3.4. Sensitivity Analysis

**Figure 5:**
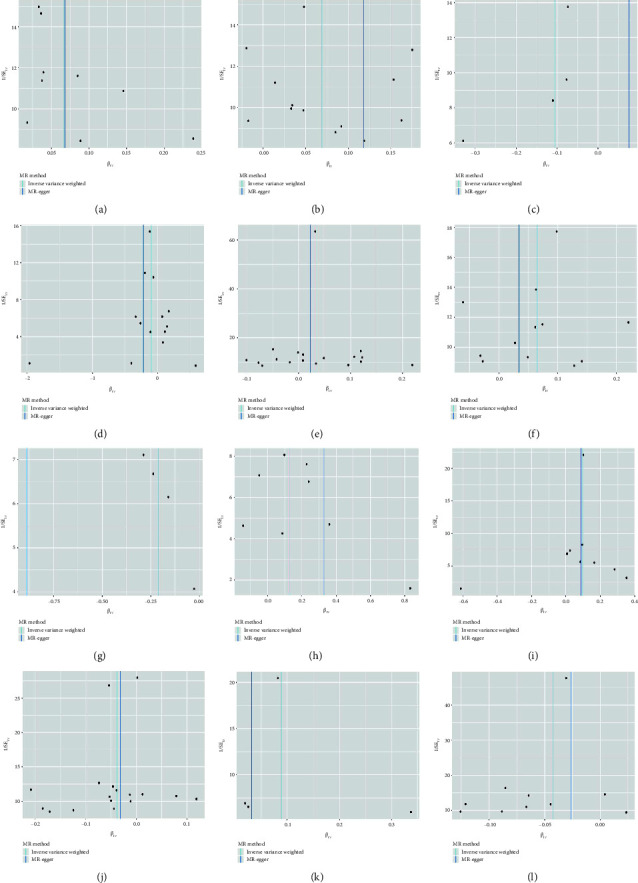
(a) Funnel plot displaying the causal association between 39S ribosomal protein L34 and breast cancer, predominantly examined utilizing the IVW method; (b) funnel plot illustrating the causal relationship between pyruvate carboxylase and breast cancer, primarily evaluated using the IVW method; (c) funnel plot depicting the causal connection between cytochrome c oxidase subunit 8A and breast cancer, predominantly assessed through the IVW method; (d) funnel plot illustrating the causal association between ADP-ribose pyrophosphatase and breast cancer, primarily examined using the IVW method; (e) funnel plot showcasing the causal relationship between rRNA methyltransferase 3 and breast cancer, predominantly evaluated through the IVW method; (f) funnel plot illustrating the causal relationship between cytochrome c oxidase assembly factor 3 homolog and breast cancer, primarily assessed using the IVW method; (g) funnel plot depicting the causal association between 39S ribosomal protein L32 and breast cancer, predominantly examined through the IVW method; (h) funnel plot illustrating the causal relationship between 39S ribosomal protein L52 and breast cancer, primarily evaluated using the IVW method; (i) funnel plot depicting the causal connection between ATP synthase subunit beta and breast cancer, primarily assessed utilizing the IVW method; (J) funnel plot illustrating the causal association between ADP-ribose pyrophosphatase and breast cancer, predominantly examined using the IVW method; (k) funnel plot showcasing the causal relationship between [pyruvate dehydrogenase (acetyl-transferring)] kinase isozyme 1 and breast cancer, primarily evaluated through the IVW method; and (l) funnel plot depicting the causal association between cytochrome c oxidase subunit 8A and breast cancer, predominantly assessed using the IVW method.

**Figure 6:**
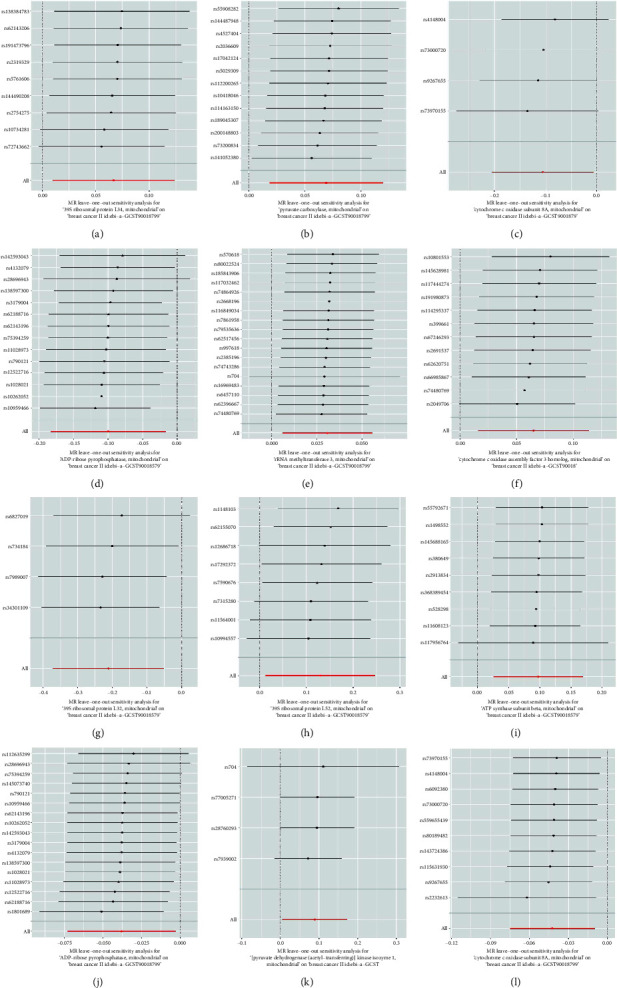
(a) Forest plot utilizing the “leave-one-out” method illustrating the causal relationship between 39S ribosomal protein L34 and breast cancer, predominantly assessed through the IVW method; (b) forest plot employing the “leave-one-out” method displaying the causal relationship between pyruvate carboxylase and breast cancer, primarily evaluated using the IVW method; (c) forest plot using the “leave-one-out” method demonstrating the causal relationship between cytochrome c oxidase subunit 8A and breast cancer, mainly assessed through the IVW method; (d) forest plot utilizing the “leave-one-out” method revealing the causal relationship between ADP-ribose pyrophosphatase and breast cancer, primarily assessed using the IVW method; (e) forest plot employing the “leave-one-out” method indicating the causal relationship between rRNA methyltransferase 3 and breast cancer, primarily evaluated using the IVW method; (f) forest plot utilizing the “leave-one-out” method illustrating the causal relationship between cytochrome c oxidase assembly factor 3 homolog and breast cancer, primarily assessed through the IVW method; (g) forest plot employing the “leave-one-out” method demonstrating the causal relationship between 39S ribosomal protein L32 and breast cancer, primarily evaluated using the IVW method; (h) forest plot using the “leave-one-out” method revealing the causal relationship between 39S ribosomal protein L52 and breast cancer, primarily assessed through the IVW method; (i) forest plot employing the “leave-one-out” method illustrating the causal relationship between ATP synthase subunit beta and breast cancer, primarily evaluated using the IVW method; (j) forest plot utilizing the “leave-one-out” method indicating the causal relationship between ADP-ribose pyrophosphatase and breast cancer, primarily assessed using the IVW method; (k) forest plot employing the “leave-one-out” method demonstrating the causal relationship between [pyruvate dehydrogenase (acetyl-transferring)] kinase isozyme 1 and breast cancer, primarily evaluated through the IVW method; and (l) forest plot utilizing the “leave-one-out” method illustrating the causal relationship between cytochrome c oxidase subunit 8A and breast cancer, primarily assessed using the IVW method.

Both the MR-Egger regression and IVW methods revealed no heterogeneity across all datasets, with calculated results indicating *p* values greater than 0.05. The funnel plot displayed a symmetrical distribution of SNPs, signifying minimal bias and indicating the stability and reliability of the results (Figure 5). To delve deeper into the potential presence of directed level multidirectionality, Egger regression directionality was conducted. The analysis confirmed the absence of directed level multidirectionality across all datasets (Figure 6 and Table 6).

## 4. Discussion

In this research endeavor, we leveraged extensive public GWAS data and conducted MR analyses in R to delve into the potential causal correlation between mitochondrial function and breast cancer. The outcomes of our investigation unveiled various mitochondrial genes that exhibit association with both risk-enhancing and protective roles across these diverse populations. Notably, within the European cohort, 39S ribosomal protein L34, pyruvate carboxylase, rRNA methyltransferase 3, and cytochrome c oxidase assembly factor 3 homolog emerged as risk factors, while cytochrome c oxidase subunit 8A and ADP-ribose pyrophosphatase were identified as protective factors. Conversely, in the East Asian population, 39S ribosomal protein L52, ATP synthase subunit beta, and [pyruvate dehydrogenase (acetyl-transferring)] kinase isozyme 1 were delineated as risk factors, with 39S ribosomal protein L32, ADP-ribose pyrophosphatase, and cytochrome c oxidase subunit 8A recognized as protective elements.

The analysis unveiled a direct association between 39S ribosomal protein L33 and ER+ breast cancer, identifying it as a protective factor (IVW, *p*=0.0015, OR = 0.965, 95% CI: 0.943–0.986). *N*-Acetylglutamate synthase was recognized as a risk factor causally linked to ER+ breast cancer (IVW, *p*=0.025, OR = 1.060, 95% CI: 1.008–1.116). In addition, NADH dehydrogenase [ubiquinone] iron-sulfur protein 4 was found to be causally associated with ER+ breast cancer as a risk factor (IVW, *p*=0.031, OR = 1.017, 95% CI: 1.002–1.033). Moreover, [pyruvate dehydrogenase (acetyl-transferring)] kinase isozyme 1 emerged as a protective factor against ER+ breast cancer (IVW, *p*=0.0078, OR = 0.960, 95% CI: 0.931–0.989).

For ER− breast cancer, hydroxymethylglutaryl-CoA synthase emerged as a causative factor associated with breast cancer, acting as a protective element (IVW, *p*=0.044, OR = 0.935, 95% CI: 0.875–0.998). Conversely, mitochondrial glutamate carrier 2 was found to be causally linked to ER− breast cancer as a risk factor (IVW, *p*=0.049, OR = 1.083, 95% CI: 1.000–1.174), alongside mitochondrial sodium/hydrogen exchanger 9B2 identified as a risk factor for ER− breast cancer (IVW, *p*=0.031, OR = 1.074, 95% CI: 1.006–1.145). Essential MCU regulator was also causally associated with ER− breast cancer as a risk factor (IVW, *p*=0.0077, OR = 1.071, 95% CI: 1.018–1.127). In contrast, the ES1 protein homolog emerged as a protective factor against breast cancer (IVW, *p*=0.035, OR = 0.943, 95% CI: 0.893–0.996). The mitochondrial import inner membrane translocase subunit TIM14 was identified as a risk factor causally linked to ER– breast cancer (IVW, *p*=0.045, OR = 1.071, 95% CI: 1.002–1.145).

Mitochondria serve as the primary locus for ATP generation in healthy cells through oxidative phosphorylation, playing a fundamental role in powering energy-dependent processes and anabolic functions [21]. In addition, they play a crucial role in tumorigenesis by providing essential metabolic substrates. Maintaining their structural and functional integrity, mitochondria undergo a perpetual cycle of fusion and fission under normal physiological conditions [22]. Past studies have underscored the significant involvement of mitochondria in the progression of breast cancer, with therapeutic strategies targeting mitochondrial function demonstrating promise in impeding cancer advancement [23, 24].

In line with our discoveries, current literature corroborates our findings. Recent studies have underscored the significance of mitochondrial genes, including 39S ribosomal proteins, pyruvate carboxylase, and cytochrome c oxidase subunits, in the development of breast cancer [25, 26]. These genes play pivotal roles in mitochondrial functions governing energy metabolism and responses to oxidative stress, ultimately impacting the predisposition to cancer.

Our investigation delves into the variance in mitochondrial pathways between European and East Asian populations, accentuating the genetic heterogeneity in susceptibility to cancer. Studies centered on these population groups underscore the role of mitochondrial anomalies in the genesis of breast cancer [27, 28]. The genetic alterations pinpointed in our analysis, such as ATP synthase subunit beta and [pyruvate dehydrogenase (acetyl-transferring)] kinase isozyme 1, underscore distinctive risk elements specific to each population [29, 30].

While our research primarily centers on statistical methodologies, experimental data substantiates the critical role of mitochondrial function in cancer biology. Empirical studies have showcased that interventions targeting mitochondrial pathways can inhibit tumor proliferation and enhance treatment responses in breast cancer and various cancer types [31, 32]. These observations underscore the clinical significance of our MR approach in pinpointing plausible therapeutic targets.

Our study's strengths encompass a meticulous examination of GWAS data and the application of rigorous statistical techniques to mitigate biases, including thorough correlation and LD analyses, in addition to employing stringent criteria for SNP selection. The symmetrical arrangement of SNPs in funnel plots, along with the consistency validated by numerous robustness assessments and leave-one-out analyses, reinforce the credibility and dependability of our findings.

This study is subject to several limitations as follows: (1) despite the absence of significant heterogeneity indicated by MR-Egger regression and the IVW method, potential sources of heterogeneity may stem from variations in analysis platforms and IVs utilized across diverse experiments and populations, necessitating further exploration; (2) the restricted number of IVs for SNPs in each dataset suggests the requirement for larger sample sizes to identify additional SNPs for more comprehensive analyses. Enlarging the sample size could enhance the study's statistical power and strengthen the validity of the results; and (3) while the analysis focused on assessing the causal relationship between mitochondrial function and breast cancer through MR, the investigation did not delve into the underlying mechanisms [33].

## Figures and Tables

**Table 1:** Information on GWAS data related to breast cancer.

GWAS-ID	Name	Population	Sample	SNPs
ebi-a-GCST90018799	Breast cancer	European	257,730	24,133,589
ebi-a-GCST90018579	Breast cancer	East Asian	79,550	12,429,464
ieu-a-1127	ER+ breast cancer	European	175,475	10,680,257
ieu-a-1128	ER− breast cancer	European	127,442	10,680,257

**Table 2:** Mitochondrial-related GWAS data information.

GWAS-ID	Name	Sample	SNPs
prot-a-1943	39S ribosomal protein L34, mitochondrial	3301	24,133,589
prot-a-2190	Pyruvate carboxylase, mitochondrial	3301	24,133,589
prot-a-641	Cytochrome c oxidase subunit 8A, mitochondrial	3301	24,133,589
prot-a-2129	ADP-ribose pyrophosphatase, mitochondrial	3301	24,133,589
prot-a-2575	rRNA methyltransferase 3, mitochondrial	3301	24,133,589
prot-a-612	Cytochrome c oxidase assembly factor 3 homolog, mitochondrial	3301	24,133,589
prot-a-1941	NADH dehydrogenase [ubiquinone] 39S ribosomal protein L32, mitochondrial	3301	12,429,464
prot-a-1944	39S ribosomal protein L52, mitochondrial	3301	12,429,464
prot-a-203	ATP synthase subunit beta, mitochondrial	3301	12,429,464
prot-a-2129	ADP-ribose pyrophosphatase, mitochondrial	3301	12,429,464
prot-a-2235	[Pyruvate dehydrogenase (acetyl-transferring)] kinase isozyme 1, mitochondrial	3301	12,429,464
prot-a-641	Cytochrome c oxidase subunit 8A, mitochondrial	3301	12,429,464

**Table 3:** Mitochondrial dataset tool variation scale.

GWAS-ID	Type	Population	SNPs
prot-a-1943	Breast cancer	European	9
prot-a-2190	Breast cancer	European	13
prot-a-2575	Breast cancer	European	18
prot-a-612	Breast cancer	European	12
prot-a-1941	Breast cancer	East Asian	4
prot-a-1944	Breast cancer	East Asian	8
prot-a-203	Breast cancer	East Asian	10
prot-a-2235	Breast cancer	East Asian	4
prot-a-2129	Breast cancer	European	17
		East Asian	14
prot-a-641	Breast cancer	European	10
		East Asian	4

**Table 4:** Summary of SNPs acting as risk or protective factors for breast cancer.

Population	GWAS-ID	Name	*p* value	OR	95% CI	Risk/protective
European	prot-a-1943	39S ribosomal protein L34, mitochondrial	0.021	1.069	1.010–1.132	Risk
European	prot-a-2190	Pyruvate carboxylase, mitochondrial	0.007	1.071	1.019–1.127	Risk
European	prot-a-641	Cytochrome c oxidase subunit 8A, mitochondrial	0.059	0.963	0.928–0.990	Protective
European	prot-a-2129	ADP-ribose pyrophosphatase, mitochondrial	0.011	0.962	0.930–0.997	Protective
European	prot-a-2575	rRNA methyltransferase 3, mitochondrial	0.015	1.031	1.006–1.057	Risk
European	prot-a-612	Cytochrome c oxidase assembly factor 3 homolog, mitochondrial	0.009	1.067	1.016–1.121	Risk
East Asian	prot-a-1941	39S ribosomal protein L32, mitochondrial	0.010	0.809	0.690–0.950	Protective
East Asian	prot-a-1944	39S ribosomal protein L52, mitochondrial	0.029	1.138	1.013–1.279	Risk
East Asian	prot-a-203	ATP synthase subunit beta, mitochondrial	0.048	1.084	1.027–1.184	Risk
East Asian	prot-a-2129	ADP-ribose pyrophosphatase, mitochondrial	0.019	0.905	0.833–0.983	Protective
East Asian	prot-a-2235	[Pyruvate dehydrogenase (acetyl-transferring)] kinase isozyme 1, mitochondrial	0.038	1.093	1.005–1.189	Risk
East Asian	prot-a-641	Cytochrome c oxidase subunit 8A, mitochondrial	0.037	0.900	0.815–0.994	Protective

**Table 5:** MR analysis of mitochondria and breast cancer subtypes.

Type	Id	Name	Snp	OR (95% CI)	*p* value
The ER+ breast cancer	prot-a-1942	39S ribosomal protein L33	11	0.965 (0.943–0.986)	1.5*e* − 03
The ER+ breast cancer	prot-a-1997	*N*-Acetylglutamate synthase	7	1.060 (1.008–1.116)	2.5*e* − 02
The ER+ breast cancer	prot-a-2025	NADH dehydrogenase [ubiquinone] iron-sulfur protein 4	19	1.017 (1.002–1.033)	3.1*e* − 02
The ER+ breast cancer	prot-a-2235	[Pyruvate dehydrogenase (acetyl-transferring)] kinase isozyme 1	9	0.960 (0.931–0.989)	7.8*e* − 03
The ER− breast cancer	prot-a-1356	Hydroxymethylglutaryl-CoA synthase	11	0.935 (0.875–0.998)	4.4*e* − 02
The ER− breast cancer	prot-a-2749	Mitochondrial glutamate carrier 2	13	1.083 (1.000–1.174)	4.9*e* − 02
The ER− breast cancer	prot-a-2764	Mitochondrial sodium/hydrogen exchanger 9B2	9	1.074 (1.006–1.145)	3.1*e* − 02
The ER− breast cancer	prot-a-2776	Essential MCU regulator	15	1.071 (1.018–1.127)	7.7e−03
The ER− breast cancer	prot-a-308	ES1 protein homolog	14	0.943 (0.893–0.996)	3.5*e* − 02
The ER− breast cancer	prot-a-847	Mitochondrial import inner membrane translocase subunit TIM14	9	1.071 (1.002–1.145)	4.5*e* − 02

**Table 6:** Heterogeneity analysis.

GWAS-ID	Population	Heterogeneity tests		Directional horizontal pleiotropy tests
		MR-Egger	IVW	
prot-a-1943	European	0.795	0.869	0.984
prot-a-2190	European	0.871	0.888	0.505
prot-a-641	European	0.944	0.940	0.428
prot-a-2129	European	0.541	0.609	0.810
prot-a-2575	European	0.588	0.640	0.641
prot-a-612	European	0.595	0.638	0.505
prot-a-1941	East Asian	0.832	0.807	0.517
prot-a-1944	East Asian	0.399	0.450	0.482
prot-a-203	East Asian	0.852	0.909	0.856
prot-a-2129	East Asian	0.363	0.237	0.114
prot-a-2235	East Asian	0.376	0.456	0.505
prot-a-641	East Asian	0.837	0.541	0.312

## Data Availability

All data are available from the following website: https://gwas.mrcieu.ac.uk/.

## Supporting Information

Supporting Information Tables of the main results of the MR Analysis.
